# Intelligent Optimization Control of Plate Plan-View Pattern Based on Intermediate Slab Pattern Vision Inspection and BWO-DNN Algorithm

**DOI:** 10.3390/ma18133038

**Published:** 2025-06-26

**Authors:** Zhong Zhao, Chujie Liu, Jiawei Wang, Junyi Luo, Zhiqiang Wang, Zhiqiang Wu, Chunyu He, Zhijie Jiao

**Affiliations:** State Key Laboratory of Digital Steel, Northeastern University, Shenyang 110819, China; zhaozhong@ral.neu.edu.cn (Z.Z.); liu17837452251@163.com (C.L.); wjw2289824938@163.com (J.W.); luojuny0617@163.com (J.L.); wangzhiq1229@163.com (Z.W.); wuzq@ral.neu.edu.cn (Z.W.)

**Keywords:** plate, PVPC, intermediate slab, machine learning, BWO-DNN

## Abstract

In the production process of plate, the main factors affecting the yield of plate are the crop-cutting and edge losses. It is very important to accurately predict the crop pattern of plates and effectively control the plan-view pattern. In this paper, a detection scheme is proposed to obtain the plan-view pattern of the intermediate slab and finished plate by placing detection devices after the roughing mill and finishing mill, respectively. An image processing algorithm is used to obtain a dataset of the plan-view pattern parameters, and a plan-view pattern prediction and control model for plate is established based on the BWO-DNN (beluga whale optimization–deep neural network) algorithm. The BWO algorithm is used to optimize the hyperparameters in the DNN algorithm to complete the establishment of the intelligent model. In terms of model analysis, goodness of fit (R^2^) and mean absolute error (MAE) are used as evaluation indicators. The results show that the intelligence model established based on BWO-DNN has good predictive and control performance, realizing intelligent prediction of the crop pattern of plates and parameter optimization of plan-view pattern control. The actual production verification on site shows that the irregular areas of the plate head and tail can be reduced by 17.2% and 22.6%, respectively.

## 1. Introduction

**Figure 1:**
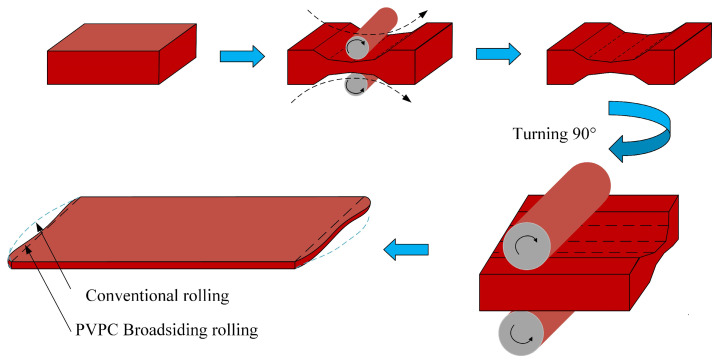
Rolling process of PVPC [3].

Plates, as important steel materials, are widely used in infrastructure construction and mechanical manufacturing [1]. The yield of plates is an important economic and technical indicator for evaluating enterprises’ resource utilization and competitiveness. The main factor influencing the yield is the shear loss caused by the restriction of the plan-view pattern [2]. Plate plan-view pattern control (PVPC) is a very effective method to make the plate rectangular, reduce crop and edge losses, and improve the yield. Its basic idea is to predict the pattern of the rolled piece after rolling, and convert the volume of the irregular pattern into an abnormal thickness compensation amount, given in the last pass of the sizing and broadening phase according to the “constant volume principle”. This abnormal thickness distribution is used to improve the final rectangularity. The basic principle of plan-view pattern control is shown in Figure 1.

Extensive research has been conducted by scholars on plan-view pattern control. Early studies primarily adopted mechanism models, deriving theoretical equations for three-dimensional metal flow and spreading through basic laws such as the equal volume method and the minimum resistance method. These models provided a foundational basis for analyzing the plan-view pattern of plates. For example, Ding [4] established a mathematical model for the plan-view pattern of plates to explore the variation law of deformation parameters during the rolling process. Liu [5] used the energy method to develop an accurate prediction model for plate rolling. However, mechanism models often exhibit substantial errors due to the simplification and assumptions of physical phenomena. In contrast, the finite element analysis method can accurately predict stress, strain, and temperature distributions through fine discretization, adapting to material nonlinearity and complex boundary conditions. This not only enhances the accuracy of plan-view pattern control but also provides more reliable support for optimizing process parameters.

Hu and Zhang [6,7] established plan-view pattern control models for plates based on simulation and proposed new technologies such as the TFP (trimming-free process) to improve slab yield. Xun [8] employed the elasto-plastic finite element method to analyze the influence of rolling conditions on the plate plan-view pattern, proposing a calculation formula for the plan-view pattern curve. Yang et al. [9] established a plan-view pattern prediction model based on the longitudinal length difference at the plate head and tail, improving prediction accuracy through the relationship between the metal volume and length difference, and verified the model’s effectiveness through finite element simulation and experiments. Ding et al. [10] studied the influence of setting points and distances on the rectangularity of products using a controllable point setting method combined with finite element analysis, successfully increasing the production yield from 92.28% to 93.36%. Yin [11] analyzed metal flow through simulating the rolling process, compared the plan-view patterns under different rolling schedules, and obtained a comprehensive optimal rolling schedule. Masayuki Horie [12] et al. studied the dog-bone rolling method and found that the dog-bone width and broadening ratio significantly influence the plastic deformation length and fish-tail length. When the width increases or the broadening ratio decreases, the fish-tail length increases. Gu [13] used the finite element method to study the control of the plate plan-view pattern by flat and vertical rolls, proposed a theoretical model, and the simulation results matched the actual situation, reducing the difference in width elongation and effectively controlling the head’s convex shape. Wang [14] analyzed the variation law of the plate plan-view pattern through finite element modeling, and improved the width accuracy by 27% after parameter optimization. Yao [15] et al. established a prediction model and control model for plan-view pattern control of wide plates based on the constant volume law, and applied the models on site, reducing the shear loss of products.

With the rapid development of intelligent technologies, research on plate plan-view patterns has been integrated with advanced technologies such as machine learning and machine vision. Machine vision detection technology [16] is rapidly emerging in the industrial field, gradually replacing human eyes for measurement and judgment. Chen et al. [17] proposed a new strip-shape recognition method, optimizing neural networks with orthogonal polynomials and binary trees to construct a hybrid model and improve recognition accuracy. Ma [18] studied the selection of industrial cameras and image processing algorithms to realize online real-time recognition of plate contours and accurate positioning of crop positions for the head and tail. He et al. [19] used improved image processing algorithms based on machine vision technology to detect position and angle information in real time during the steel-turning process, enabling real-time processing of detection data in complex production environments. Ding et al. [20] employed machine vision to measure the camber of plates, obtaining sub-pixel coordinates of the rolled piece edges and determining their plan dimensions to implement feedback control for camber defects. Yang [21] achieved high-precision recognition and contour feature extraction of plates based on machine vision technologies such as binocular multi-group linear-array cameras.

Machine vision technology combined with machine learning algorithms can analyze and process massive data and image information to realize monitoring, prediction, correction, and optimization of plate deformation, defects, and plan-view patterns. Machine learning models aim to tap the potential in feature extraction and model construction for plate plan-view patterns, deepening the intelligent research on plan-view pattern control. Wang [22,23] built a BP (back-propagation) neural network to establish metal rolling flow prediction models, though faced challenges such as data deviation and long computation times. M.S. Chun [24] established a multi-layer neural network model to predict width changes during plate rolling, determining the optimal broadening value to reduce edge-trimming losses. Dong [25] developed an ISSA-ANN (improved sparrow search algorithm–artificial neural network) plan-view pattern prediction model based on on-site production data, optimizing initial weights and thresholds to overcome the problem that the traditional neural network easily produced local optima. Jiao et al. [3] optimized a radial basis function neural network by using the dung beetle optimizer algorithm, and developed a prediction and control model for plate plan-view pattern. In practical on-site applications, the crop-cutting loss area of irregular deformation in the plate was reduced by 31%. Wu et al. [26] proposed an attention-based weight-adaptive multi-task learning framework for predicting and optimizing irregular shapes in hot rolling, optimizing the short-stroke process to achieve a 10.3% improvement in defect width deviation. Zhao [27] studied the application of extreme learning machines in predicting the length of rolled pieces, optimizing the prediction model for the plate plan-view pattern.

Currently, research work on intelligent optimal control of the plate plan-view pattern using machine vision inspection is based on pattern inspection of finished plates at the end of rolling. However, passes for plan-view pattern control occur in the rough-rolling stage, specifically at the final pass of the sizing phase and the broadsiding phase. Due to the requirements of the thermo-mechanically controlled processing (TMCP) rolling process for plates, there is a holding temperature process after the rough-rolling stage. Multiple rolled pieces first complete the rough-rolling stage and holding temperature process before proceeding to subsequent rolling passes to become products. Therefore, product pattern-detection results cannot be followed by rolled pieces for real-time correction, i.e., plan-view pattern control optimization has a significant hysteresis. This hysteresis not only reduces the accuracy and efficiency of plan-view pattern control for plates, but also may lead to resource waste and increased production costs. If an accurate description of the final shape can be obtained immediately after the end of rough rolling, the plan-view pattern of the next piece can be optimized and adjusted in good time.

Based on the above background, by reasonably arranging detection devices, the intermediate slab plan-view pattern of a rolled piece can be obtained after rough rolling. Then, according to the subsequent rolling process conditions, machine learning algorithms can be used to predict the final shape in advance, enabling timely feedback and optimization of plan-view pattern control for the next rolled piece. This approach demonstrates significant advantages over traditional finished-product inspection models, notably improving production response speed and reducing rejection rates caused by delayed parameter adjustment. Existing actual data from product shape detection serves as both a data source for machine learning for a model predicting the final pattern from the intermediate pattern, and an evaluation tool for the final control effect.

In the research process, a typical two-stand plate production line is taken as the research object, deploying visual detection devices after both the rough mill and the finish mill to collect intermediate slab and final plate plan-view pattern data during rolling. Using intermediate slab pattern data and the subsequent rolling schedule data as inputs, the corresponding final pattern prediction is obtained through a machine learning algorithm as the output result. Based on the final shape prediction, the plan-view pattern control effect is evaluated and the control parameters are optimized, and the plan-view pattern of the subsequent rolled piece is optimized in real time. The actual measurement data of the final plate pattern serves as the ultimate evaluation for the prediction and control models.

## 2. Data Acquisition

### 2.1. Industrial Camera Settings

**Figure 2:**
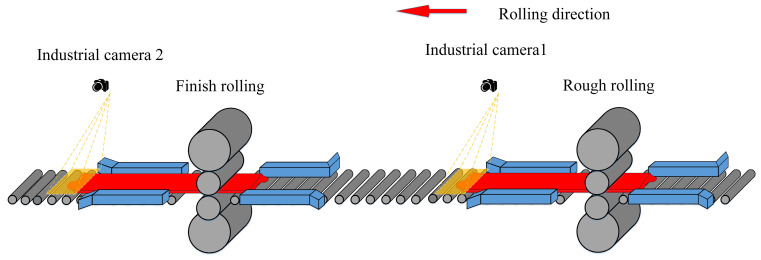
Industrial camera installation position.

In order to obtain plan-view pattern measured data of the intermediate slab and plate, industrial cameras were installed at the exits of the rough-rolling mill and the finish-rolling mill for image acquisition, as shown in Figure 2. Industrial camera 1 was installed at the exit of the rough-rolling mill to collect the plan-view pattern image of the intermediate slab, and industrial camera 2 collects the plan-view pattern image of the finished plate.

### 2.2. Intermediate Slab Detection

**Figure 3:**
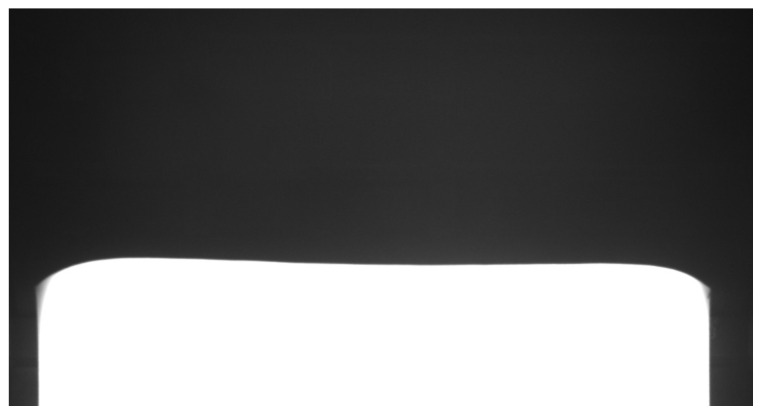
Intermediate slab head gray image.

After the rough-rolling stage, PVPC pass-rolling was completed. The plan-view pattern of the plate can be predicted in advance by using the plan-view pattern of the intermediate slab and the subsequent rolling schedule. The image of the intermediate slab’s head and tail after rough rolling was collected by the industrial camera, and the image of the intermediate slab was grayed, and then projection transformation was performed to obtain a gray image from the top-view angle, as shown in Figure 3. This operation was convenient for subsequent better identification of the intermediate slab head and tail and extracted the characteristics of the intermediate slab.

**Figure 4:**
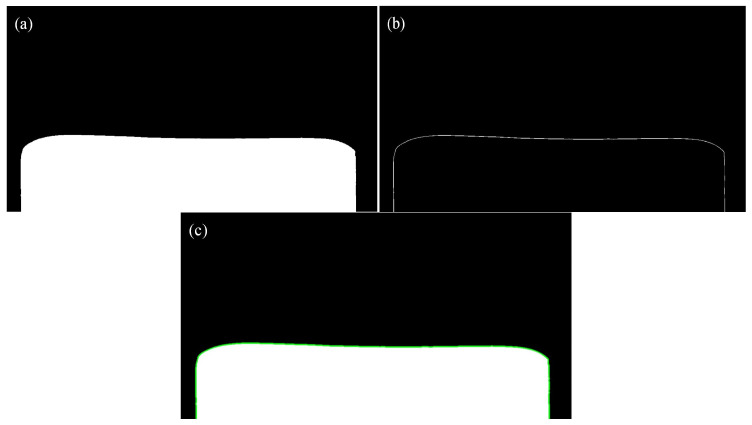
Intermediate billet image processing flow: (**a**) binarization; (**b**) edge detection; (**c**) contour extraction.

In order to more accurately find the intermediate slab and plate’s head and tail shear lines, it is necessary to obtain accurate and clear images first. Due to the different temperatures of plates in different passes and the different light and darkness of the workshop at different times, the brightness of images is affected, and it is not possible to accurately segment each plate image, which in turn affects the extraction of the plate profile. Moreover, the harsh environment in the rolling site, with issues such as water vapor, dust, and light interference, affects the clarity of images acquired by the measuring device. Therefore, image processing algorithms need to be adopted to process the collected images. The gray image of the intermediate slab after projection transformation is binarized by an adaptive threshold adjustment plate image thresholding method combined with Gamma image enhancement and the Canny edge extraction algorithm, and then the profile of the intermediate slab’s head and tail is extracted. The overall process is shown in Figure 4.

### 2.3. Plate Profile Coordinate Point Processing

**Figure 5:**
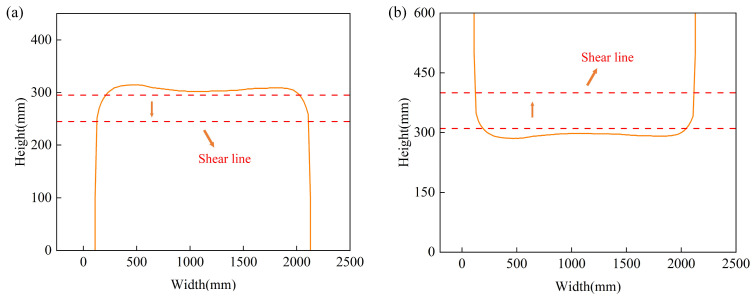
Determination of the best shear line: (**a**) head; (**b**) tail.

The image of the plate taken by the industrial camera is not just of the head and tail irregular area that needs to be cut, so it is necessary to further segment the head and tail edge profile of the processed plate. The contour pixel data is globally traversed and the pixels with the same Y value on both sides are connected. As shown in Figure 5, the image is scanned from top to bottom, the connection line between the two points whose width is not shrinking or changing is found, as the optimal shear line, and then the coordinates of the segmented head and tail contour points are extracted.

When the computer collects the image contour pixel data, the upper left corner of the default image is the coordinate origin; so, first of all, it is necessary to transform the coordinate origin of the plate head and tail pixel coordinates. Since the starting position is randomly generated when extracting the coordinate points and the points are not averaged, in this study, the missing points are interpolated, after which the pixel points are transformed to the actual coordinates according to the roller table drawing size. In order to facilitate statistics and data management, the contour data is equidistantly processed. On the premise of ensuring fitting accuracy, 101 point coordinates are used to represent the intermediate slab profile and the plate profile.

### 2.4. Data Pre-Processing

**Figure 6:**
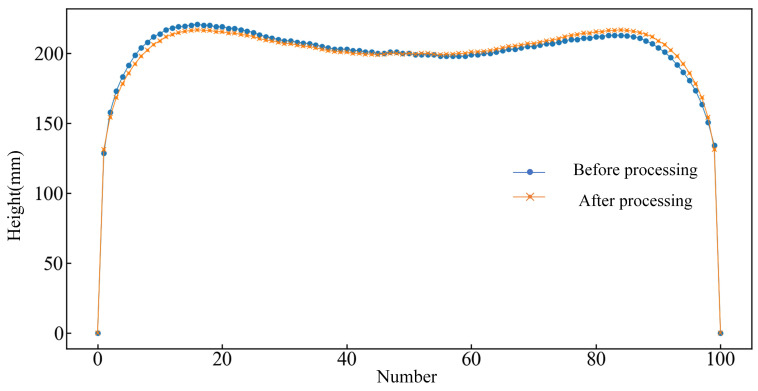
Outline point coordinate processing diagram.

In the actual rolling process, the head and tail will show irregular asymmetry, which is due to lateral asymmetric factors in the plate rolling process, such as differences in the stiffness of the two sides of the mill, plate transverse temperature inhomogeneities, as well as the plate centerline offset in the rolling process, and so on. Therefore, in the actual collection of the 101 contour points in the head–tail deformation region, with the center point as a benchmark, the left and right symmetry of the two y-value points is determined for the average processing, as shown in Figure 6. The symmetry ratio is defined as the area ratio between the left and right sides with respect to the midpoint. Through testing, it was found that the samples with poor symmetry between the plate head and tail had a great influence on the prediction performance of the intelligent model. Therefore, data samples with head and tail symmetry rates between 0.95 and 1.05 were selected.

**Figure 7:**
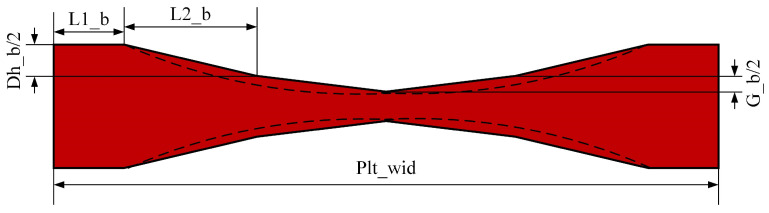
PVPC diagram.

In this paper, actual rolling data of plates produced from plants were selected as samples for the plate plan-view pattern neural network prediction model. Since theoretical control of the plan-view pattern is very complicated, in the actual rolling process the simplified model curve shown in Figure 7 is adopted for guaranteeing the control accuracy. The width center of the rolled piece is symmetrically distributed along the width direction of the plate. The control parameters are the length of the head–tail platform section L1_b, the unsteady length L2_b, the PVPC height Dh_b, and the PVPC height added value G_b.

Considering the physical model and the actual production conditions, 13 main characteristics of plates were selected, the contour points of the head and tail of the plates and the contour points of the intermediate slab were combined as the dataset for establishing the machine learning model, as shown in Table 1.

## 3. Research Method

### 3.1. Deep Neural Network

**Figure 8:**
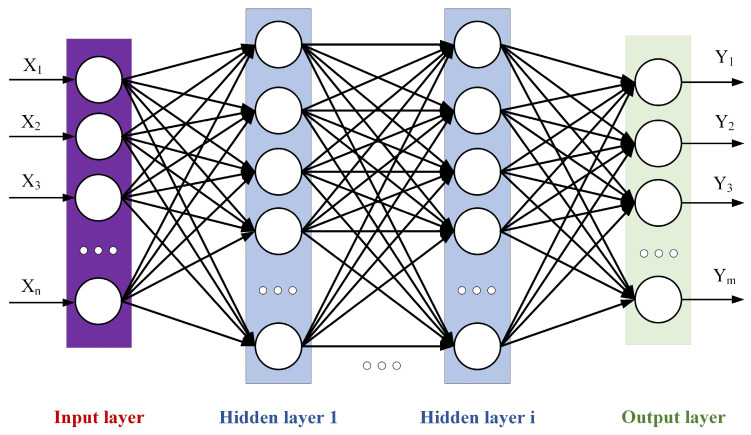
DNN neural network diagram.

A deep neural network (DNN) is an artificial neural network with multiple hidden layers [28,29], sometimes called a Multi-Layer Perceptron (MLP). Compared with traditional shallow neural networks, a DNN can learn more complex feature representations and is suitable for various complex tasks. The training process of a DNN can be parallelly calculated by hardware such as GPUs, which greatly accelerates the training speed. A DNN is divided according to the location of different layers. The internal neural network layer can be divided into three categories: the input layer, hidden layer, and output layer. As shown in Figure 8, the first layer is the input layer, the last layer is the output layer, and all the neurons in the middle are hidden-layer neurons.

In the figure, X represents the input feature, Y denotes the output feature, *n* is the number of nodes in the input layer, and m is the number of nodes in the output layer, with neurons of the hidden layer in between. Each neuron receives inputs from other neurons and adjusts the weights to change the influence of input features on the neuron. The model can achieve the approximation of complex functions and achieve the effect of universal approximation through the multi-layer nonlinear hidden layer. In this paper, the DNN neural network uses the PRelu activation function, as shown in Equation (1).

(1) $$PRelu\left(x\right)=\left\{\begin{array}{c} \;\;x,\;\;x\geq 0\\ ax,\;\;x<0 \end{array}\right.$$

In the above equation, x is the output of the neuron input layer. a is automatically learned during model training. The initial values are typically set as small positive numbers between 0.1 and 0.3.

### 3.2. BWO Optimization Algorithm

Beluga whale optimization (BWO) is a novel swarm intelligence optimization algorithm that is inspired by the life behaviors of beluga whales [30]. Similar to other swarm intelligence optimization algorithms, BWO includes an exploration phase and exploitation phase. In addition, the algorithm also simulates the whale fall phenomenon in the biological world, and also introduces the Levy flight strategy to enhance the global convergence of the exploitation phase.

**Figure 9:**
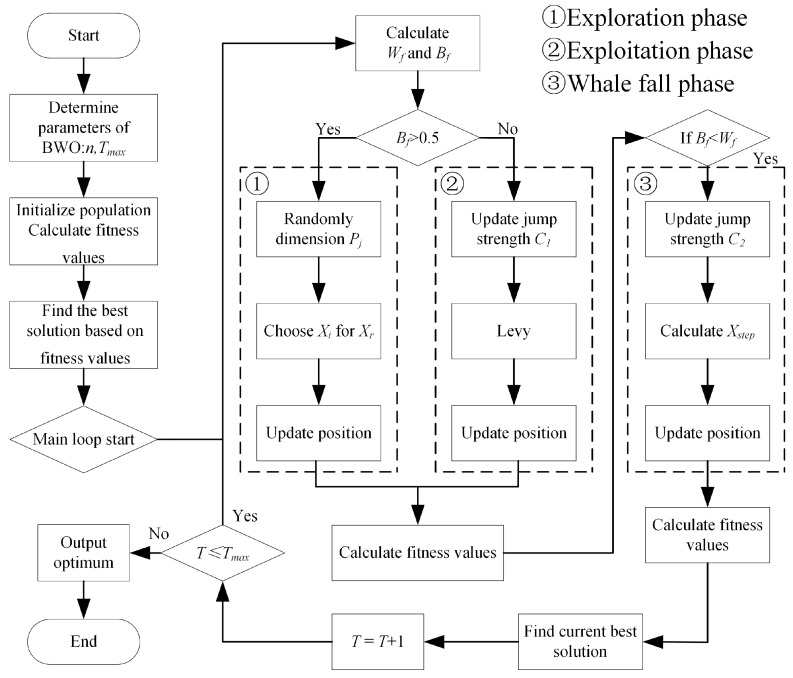
BWO algorithm flow.

The BWO algorithm first randomly initializes a set of solutions (the position of the beluga whale), and then evaluates the advantages and disadvantages of each solution through the fitness function. Then, using the predatory behavior of beluga whales, the position of each individual is dynamically updated according to the global optimal solution and the individual historical optimal solution, so as to realize the exploration and exploitation of the solution space. After several iterations, the algorithm continuously optimizes the solution until the maximum number of iterations or the fitness value is stable. Finally, the current optimal solution is output as the result. The algorithm flow chart is shown in Figure 9.

### 3.3. BWO-DNN Algorithm Design

In the DNN model, the weights and thresholds are critical to performance. However, the final weights and thresholds are constrained by the initialization, so the BWO algorithm can be used to optimize this process. The superior search performance of BWO enables the algorithm to determine the initial weights and thresholds that enable the neural network model to achieve optimal performance.

**Figure 10:**
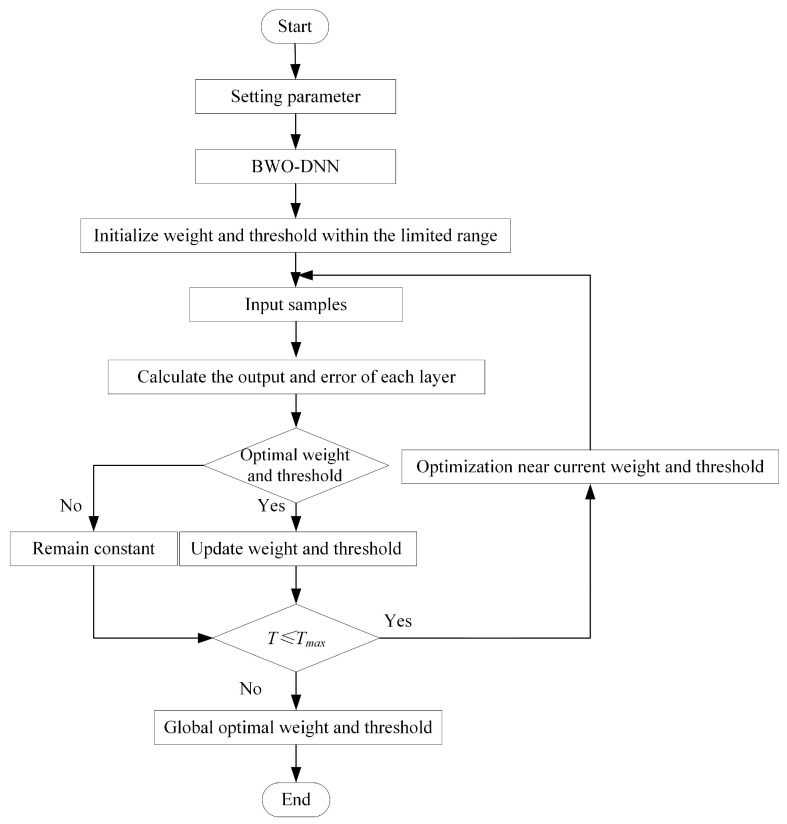
DNN-BWO algorithm flow.

A set of candidate solutions are randomly generated by the BWO algorithm. Each solution represents a set of values for the weights and thresholds. These candidate solutions are used as the initial position of the beluga whale, and then the fitness of each candidate solution is evaluated. The location is updated by simulating the predation and whale fall behaviors of beluga whale for the local and global search. The algorithm iterates until the stopping condition is satisfied, and finally outputs the optimal weight and threshold configuration to improve the performance and accuracy of the DNN model and better realize the prediction performance of the plate plan-view pattern model. The algorithm flow of BWO-DNN is shown in Figure 10.

In order to obtain a fast-converging and computationally efficient BWO-DNN model, suitable range intervals and a suitable number of populations and number of iterations need to be determined. The mean absolute error (MAE) and goodness of fit (R^2^) are used as model performance indicators for evaluation and parameter adjustment, as shown in Equations (2) and (3).

(2) $$MAE=\frac{1}{n}\left(\sum_{i=1}^{n}\left|y_{i}-y_{i}^{\prime }\right|\right)$$

(3) $$R^{2}=1-\frac{\sum_{i}{\left(y_{i}^{\prime }-\bar{y_{i}}\right)}^{2}}{\sum_{i}{\left(y_{i}-\bar{y_{i}}\right)}^{2}}$$

In the above equation, n is the number of samples in the dataset, $y_{i}$ is the actual value of the predicted variable, $y_{i}^{\prime }$ is the predicted value of the established model, and $\bar{y_{i}}$ is the mean of the sample.

According to Equations (2) and (3), it can be seen that when the mean absolute error (MAE) value is smaller, the prediction accuracy of the model is higher; the closer the goodness of fit (R^2^) is to 1, the stronger the fitting degree and interpretation ability of the prediction model on the dataset.

## 4. Results and Discussion

### 4.1. Establishment and Results of Plate Plan-View Pattern Prediction Model

**Figure 11:**
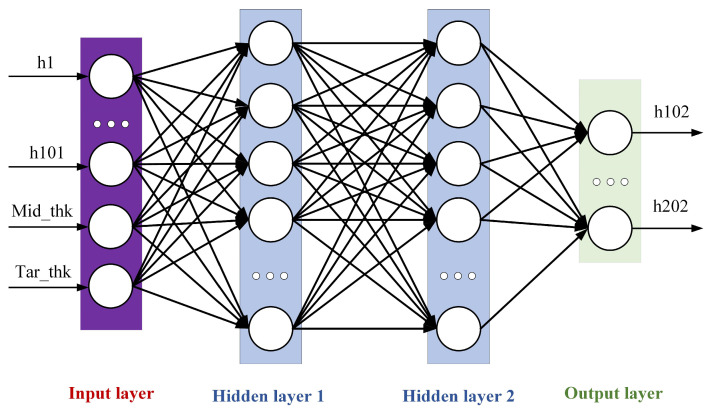
Plan-view pattern prediction model.

Considering the physical model and the actual production situation, in order to obtain a more accurate and robust plate plan-view pattern prediction model, in this study we select the coordinate Y-value of 101 contour points of the intermediate slab, the thickness of the intermediate slab, and the target thickness as the input variables of the model, and select the coordinate Y-value of 101 head (tail) contour points of the finished plate as the output variables of the model, which are combined into a dataset used to predict the plan-view pattern of the finished products from the measured plan-view pattern of the intermediate slab. The network structure of the prediction model is shown in Figure 11.

In the DNN model, an inappropriate number of hidden layers and hidden neurons can lead to overfitting or underfitting. According to the complexity of the task in this study, a neural network with a number of hidden layers between 1 and 3 and number of hidden neurons between 64 and 256 was tested to determine the optimal DNN structure, and the batch size was taken as 128. During the process of determining the optimal neural network structure, the learning rate was set to 0.0001, the dropout ratio was set to 0.2, and the optimization function was set to the Adam optimizer. Since the neural network is a multi-output model, in this study we used the average R^2^ and average MAE of the test dataset as evaluation indicators. The test results are shown in Table 2.

From the table, it can be observed that when the number of hidden layers is the same, the prediction performance of DNN improves with an increase in the number of neurons. However, when the number of neurons reaches a certain level, increasing the number of neurons instead leads to overfitting. By comparing the prediction results of different network structures, the best DNN network structure for the plan-view pattern prediction model can be identified as 103–128–256–101. After the optimal model structure was obtained, the initial weights and thresholds of the model on this basis were checked, and it was found that the range was concentrated between −0.12 and 0.12. In order to obtain the most efficient model, it is necessary to discuss the relevant parameters of BWO, so the number of populations and iterations were first determined within this range. Although increasing the number of populations and iterations can avoid the possibility of falling into a local optimal solution due to the small number of populations or failing to find the optimal solution due to the small number of iterations, if the number of populations and iterations are set too large, it may lead to slow convergence and a significant increase in calculation time. Therefore, in this study we set the same random seeds through several commonly used population size and iteration times, and tested the head plan-view pattern prediction model based on BWO-DNN to determine the most suitable matching parameters. The average R^2^ and average MAE of the test dataset of 101 output values were used as evaluation indicators. The test results are shown in Table 3.

From the prediction results in the table, when the number of populations in the BWO algorithm is set to 50 and the number of iterations is set to 150, the prediction effect is the best. In the next step, the optimal initial weight and threshold value interval are obtained by limiting the value range of the weight and threshold to obtain the optimal weight and threshold. The results of this are shown in Table 4 below.

From the results in the table, when the value interval of the weight and threshold is limited to between −0.1 and 0.1, the best prediction performance can be obtained, so the optimal solution can be obtained in this interval. Finally, it was determined that the population number of the plate plan-view pattern prediction model based on BWO-DNN should be set to 50, the number of iterations set to 150, and the value interval of the weights and thresholds set to −0.1~0.1, completing the construction of the BWO-DNN model.

**Figure 12:**
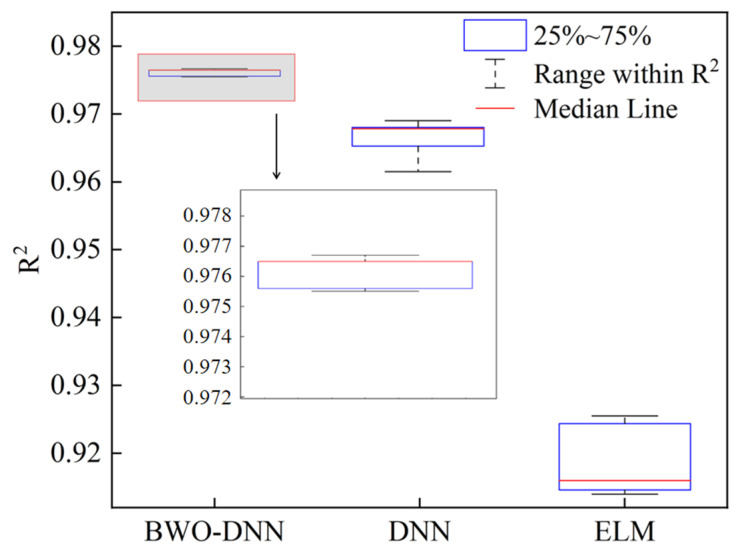
The boxplot for the R^2^ value in multiple training sessions.

Furthermore, the ELM, DNN, and BWO-DNN algorithms were used to train the plate plan-view pattern prediction model, and the prediction performance of different algorithms was compared. Figure 12 shows the distribution of R^2^ in the test dataset of the three different machine learning algorithms. Taking the median result as the stable result of prediction, it can be seen that the BWO-DNN model has a better prediction performance and a more stabilized R^2^ value compared to the ELM and DNN models.

**Figure 13:**
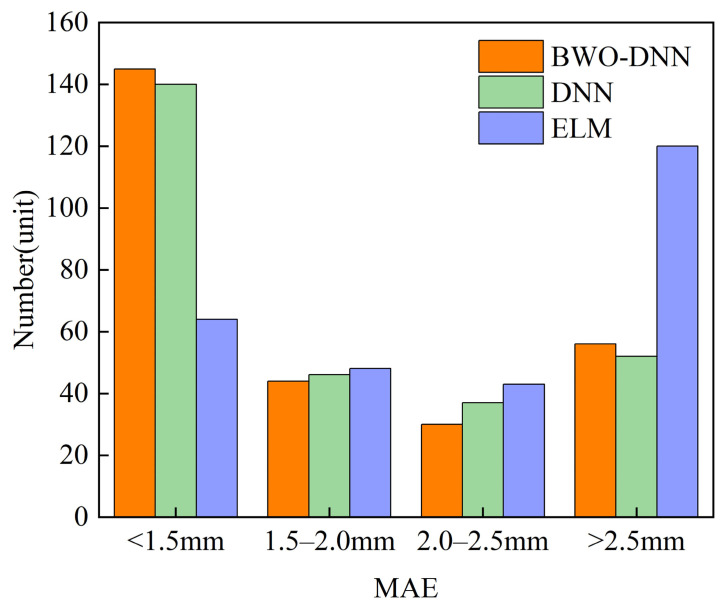
MAE distributions of different algorithms.

The MAE values of the median results of BWO-DNN, DNN, and ELM are 1.659 mm, 1.6997 mm, and 2.524 mm, respectively. Figure 13 is the MAE value distribution of the three algorithms. It can be seen from the figure that the average absolute error distribution of the prediction results of the BWO-DNN algorithm is more concentrated, and the generalization ability of the BWO-DNN algorithm is the best.

### 4.2. Establishment and Results of Plate Plan-View Pattern Control Model

**Figure 14:**
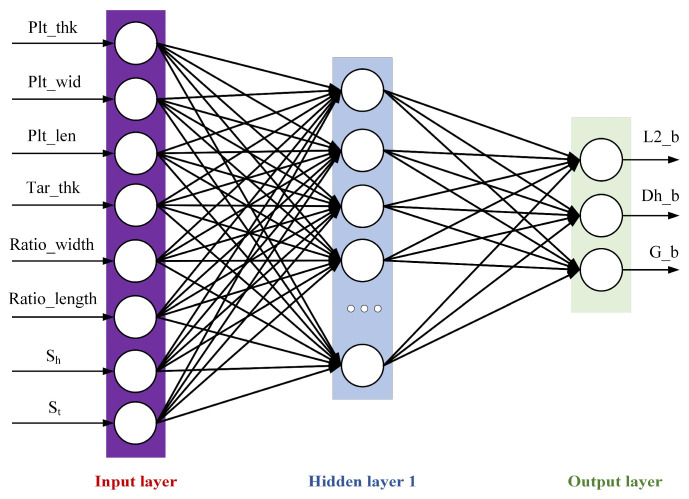
Plan-view pattern control model.

Using the plan-view pattern prediction model, the final pattern of the plate can be obtained in real-time through intermediate slab pattern detection. A neural network model was established between the irregular region of the head and tail and the PVPC parameters, and the most suitable PVPC parameters were derived through adjustment and optimization. In this study we selected eight key feature variables with significant impacts on the control parameters as input and three control parameters as output to form the dataset. The network structure of the PVPC model is shown in Figure 14.

Similar to the establishment process of the plan-view pattern prediction model, the number of hidden layers and neurons in the network was first determined. Due to the control model of the input features and output features being relatively small, the complexity is not high; the number of hidden layers chosen was between 1 and 3, and the number of hidden neurons between 16 and 128 for testing; to determine the optimal DNN structure, the batch size was taken as 128. Subsequently, the optimal population size, number of iterations, and the weights and thresholds range for BWO-DNN were determined. The results are shown in Table 5, Table 6 and Table 7.

The best DNN network structure of the plan-view pattern control model is 8-128-3, the best combination of population size and number of iterations is 30–80, and the upper and lower limits of the weights and thresholds are restricted to −0.25 and 0.25.

**Figure 15:**
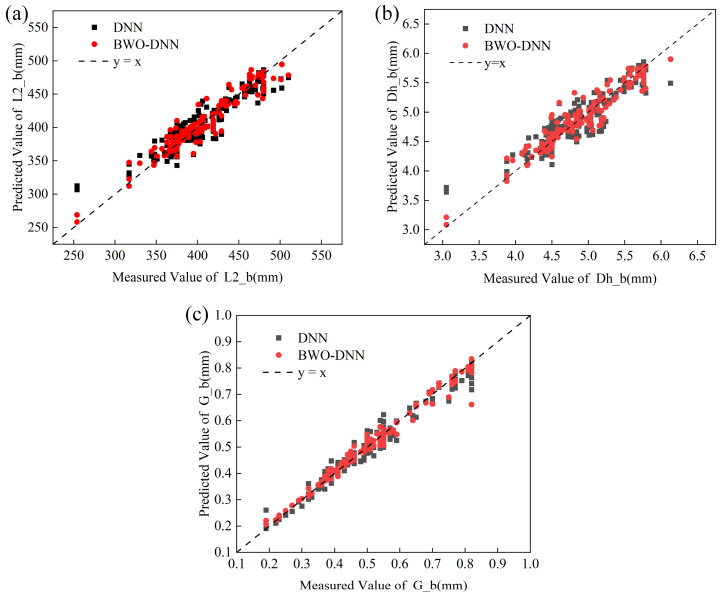
BWO-DNN and DNN prediction results: (**a**) L2_b comparison results; (**b**) Dh_b comparison results; (**c**) G_b comparison results.

Three algorithms, BWO-DNN, DNN, and ELM, were used to train the plate plan-view pattern control model. The three models were trained together after randomly shuffling the data samples each time to generate samples of the training dataset a total of seven times; the median results were selected as the stable prediction results. Figure 15 shows the scatter plots of the prediction results from the different algorithms. It can be seen from the figure that the sample results predicted by BWO-DNN are closer to the standard line. The stable prediction results of the three neural networks are summarized in Table 8, where BWO-DNN demonstrates the best predictive performance, with a median R^2^ of 0.9605 and an average MAE of 2.124 mm. The R^2^ and MAE of L2_b are 0.9471 and 6.2758 mm; the R^2^ and MAE of Dh_b are 0.9437 and 0.0839 mm; and the R^2^ and MAE of G_b are 0.9907 and 0.0109 mm.

### 4.3. Verification of Actual Production Data

The plan-view pattern intelligent model was applied in the plate production line to explore its application effect in actual production. A group of four slabs with the same specifications and process parameters from the same batch was selected, and their process parameters are listed in Table 9, and the dimensional deviation of the slabs is within ±0.1 mm. After predicting the irregular head shape using the developed plan-view pattern prediction model, the crop-cutting area was identified for further optimization.

The four slabs to be rolled were divided into two groups. The slab numbered 1-1 was rolled using the default PVPC parameters of production line, while the three slabs in the second group adopted the parameter settings from the developed PVPC intelligent model in this paper. The specific optimized parameters are shown in the following Table 10.

Using the above rolling parameters for rolling, the head–tail images of plates were collected, and contour point coordinates were extracted using the algorithm processing flow designed in Section 2 of this paper. The shape parameters of the irregular areas at the heads of four plates were obtained as shown in the following Table 11.

**Figure 16:**
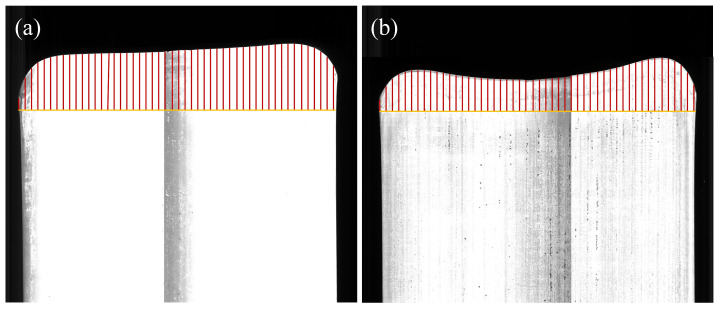
Comparison of plate plan-view pattern before and after optimization: (**a**) before optimization; (**b**) after optimization.

The data in the table show that the intelligent optimization model is significantly better than the traditional model for the head–tail irregular area. Figure 16 demonstrates the head irregular area of two plates. The colored line area in the figure shows the head irregular area of plate. The head and tail crop-cutting areas can be reduced by 17.2% and 22.6%, respectively.

## 5. Conclusions

In this paper, the plan-view pattern of intermediate slab and finished plate is detected by machine vision technology to obtain contour data. A deep neural network optimized by BWO is used to construct the intelligent optimization model of the plan-view pattern. The main results of this paper are as follows.

(1) A scheme of arranging visual inspection devices after the roughing mill and finishing mill is proposed to obtain the actual data of the intermediate slab’s plan-view pattern and the finished product’s plan-view pattern. An intelligent optimization model is used to predict the pattern of the product through the intermediate pattern data, and real-time optimization of plan-view pattern control of the next rolling piece is realized. Compared with relevant studies, the proposed new algorithm and solution significantly improve the model accuracy and reduce the head–tail shearing area.
(2) The BWO-DNN algorithm is used to construct a prediction and control model of plate plan-view pattern control, which achieves better predictions than the DNN algorithm. The product head profile was predicted, with improvement in the average R^2^ from 0.9657 to 0.9760 and the average MAE from 2.987 mm to 2.964 mm. For L2_b prediction by the intelligent control model, the R^2^ value increased from 0.9446 to 0.9471 and the MAE value decreased from 6.9873 mm to 6.2758 mm; for the prediction of Dh_b, the R^2^ value increased from 0.9139 to 0.9437 and the MAE value decreased from 0.0996 mm to 0.0839 mm. For the prediction of G_b, the R^2^ value increased from 0.9862 to 0.9907 and the MAE value decreased from 0.0122 mm to 0.0109 mm.
(3) The developed intelligent model of plate plan-view pattern control is verified in field production. Compared with the conventional model, the irregular area of the head is reduced by 17.2%. The irregular area of the tail is reduced by 22.6%.

## Figures and Tables

**Table 1:** Data feature value summary.

Index	Parameter	Description	Unit
V1	Plt_thk	Slab thickness	mm
V2	Plt_wid	Slab width	mm
V3	Plt_len	Slab length	mm
V4	Tar_thk	Target thickness	mm
V5	Ratio_width	Broadening ratio after completion of rolling	-
V6	Ratio_length	Extension ratio after completion of rolling	-
V7	L1_b	PVPC parameter	mm
V8	L2_b	PVPC parameter	mm
V9	Dh_b	PVPC parameter	mm
V10	G_b	PVPC parameter	mm
V11–V111	h1–h101	Y-value of intermediate slab contour points	mm
V112–V212	h102–h202	Y-value of plate contour points	mm
V213	Mid_thk	Intermediate slab thickness	mm
V214	S_h_	Irregular area of head	mm^2^
V215	S_t_	Irregular area of tail	mm^2^

**Table 2:** Summary of number of hidden layers and neurons for prediction model.

Number of Hidden Layers	Number of Neurons	MAE/mm	R^2^
1	128	3.823	0.9436
1	256	3.214	0.9498
2	128–128	3.566	0.9496
2	128–256	2.987	0.9657
2	256–256	3.554	0.9623
3	100–200–300	3.568	0.9532

**Table 3:** Effect of population size and iterations of BWO-DNN prediction model.

Population Size and Iterations	R^2^	MAE/mm
30–100	0.9734	2.981
30–150	0.9742	2.978
30–180	0.9750	2.977
50–100	0.9752	2.975
50–120	0.9754	2.971
50–150	0.9758	2.969
50–180	0.9758	2.969

**Table 4:** R^2^ values of prediction model with different upper and lower limits.

Value Range	R^2^	MAE/mm
−0.12~0.12	0.9758	2.969
−0.1~0.1	0.9760	2.964
−0.09~0.09	0.9760	2.964
−0.08~0.08	0.9760	2.964

**Table 5:** Summary of number of hidden layers and neurons for control model.

Number of Hidden Layers	Number of Neurons	MAE/mm	R^2^
1	32	1.621	0.9412
1	64	1.214	0.9431
1	128	1.206	0.9470
2	32–64	1.552	0.9423
2	64–128	1.571	0.9410
2	32–128	1.629	0.9399
3	32–64–128	1.680	0.9382

**Table 6:** Effect of population size and iterations of BWO-DNN control model.

Population Size and Iterations	R^2^	MAE/mm
20–50	0.9322	1.721
20–80	0.9389	1.716
20–100	0.9391	1.702
30–50	0.9426	1.627
30–80	0.9520	1.202
30–100	0.9520	1.202
50–50	0.9456	1.215

**Table 7:** R^2^ values of control model with different upper and lower limits.

Value Range	R^2^	MAE/mm
−0.30~0.30	0.9498	1.362
−0.28~0.28	0.9546	1.212
−0.25~0.25	0.9569	1.198
−0.20~0.20	0.9553	1.182
−0.18~0.18	0.9551	1.213

**Table 8:** Result comparison of different algorithms.

Parameter	Index	R^2^	MAE/mm
	BWO-DNN	0.9471	6.275
L2_b	DNN	0.9446	6.987
	ELM	0.8805	11.78
	BWO-DNN	0.9437	0.08390
Dh_b	DNN	0.9139	0.09960
	ELM	0.8583	0.1529
	BWO-DNN	0.9907	0.01090
G_b	DNN	0.9862	0.01220
	ELM	0.9631	0.01760

**Table 9:** Summary of slab data.

Parameter	Value
Material	AH36
Plt_thk/mm	220
Plt_wid/mm	2065
Plt_len/mm	2295
Tar_thk/mm	17.65
Ratio_width	1.179
Ratio_length	10.57

**Table 10:** PVPC parameter settings.

Optimization Method	Plate Number	L1_b/mm	L2_b/mm	Dh_b/mm	G_b/mm
Default parameter	1-1	100	433	5.5	0.46
Model optimization	2-1	100	538	6.51	0.82
	2-2	100	538	6.51	0.82
	2-3	100	538	6.51	0.82

**Table 11:** Measurement results of irregular areas.

Plate Number	S_h_/mm^2^	S_t_/mm^2^
1-1	1,149,151.99	871,747.05
2-1	942,693.21	690,293.28
2-2	877,396.25	661,521.32
2-3	1,034,655.85	671,400.12
Average value	951,581.77	674,404.91

## Data Availability

The original contributions presented in this study are included in the article. Further inquiries can be directed to the corresponding authors.
